# Atypical teratoid/rhabdoid tumoroids reveal subgroup-specific drug vulnerabilities

**DOI:** 10.1038/s41388-023-02681-y

**Published:** 2023-04-05

**Authors:** Irene Paassen, Justin Williams, Carla Ríos Arceo, Femke Ringnalda, Kimberly Shea Mercer, Juliane L. Buhl, Natalia Moreno, Aniello Federico, Niels E. Franke, Mariette Kranendonk, Santhosh A. Upadhyaya, Kornelius Kerl, Marc van de Wetering, Hans Clevers, Marcel Kool, Eelco W. Hoving, Martine F. Roussel, Jarno Drost

**Affiliations:** 1 Princess Máxima Center for Pediatric Oncology, Heidelberglaan 25, 3584 CS Utrecht, the Netherlands; 2 Oncode Institute, Heidelberglaan 25, 3584 CS Utrecht, the Netherlands; 3 Department of Tumor Cell Biology, St. Jude Children’s Research Hospital, Memphis, TN USA; 4 Department of Pediatric Hematology and Oncology, University Children’s Hospital Münster, Münster, Germany; 5 Hopp Children’s Cancer Center (KiTZ), 69120 Heidelberg, Germany; 6 Division of Pediatric Neurooncology, German Cancer Research Center DKFZ and German Cancer Consortium DKTK, 69120 Heidelberg, Germany; 7 Department of Oncology, St. Jude Children’s Research Hospital, Memphis, TN USA; 8 Hubrecht Institute, Royal Netherlands Academy of Arts and Sciences and University Medical Center, 3584 CT Utrecht, the Netherlands; 9 Present Address: Pharma, Research and Early Development (pRED) of F. Hoffmann-La Roche Ltd, Basel, Switzerland

**Keywords:** Cancer models, Drug screening

## Abstract

Atypical teratoid/rhabdoid tumors (ATRTs) represent a rare, but aggressive pediatric brain tumor entity. They are genetically defined by alterations in the SWI/SNF chromatin remodeling complex members *SMARCB1* or *SMARCA4*. ATRTs can be further classified in different molecular subgroups based on their epigenetic profiles. Although recent studies suggest that the different subgroups have distinct clinical features, subgroup-specific treatment regimens have not been developed thus far. This is hampered by the lack of pre-clinical in vitro models representative of the different molecular subgroups. Here, we describe the establishment of ATRT tumoroid models from the ATRT-MYC and ATRT-SHH subgroups. We demonstrate that ATRT tumoroids retain subgroup-specific epigenetic and gene expression profiles. High throughput drug screens on our ATRT tumoroids revealed distinct drug sensitivities between and within ATRT-MYC and ATRT-SHH subgroups. Whereas ATRT-MYC universally displayed high sensitivity to multi-targeted tyrosine kinase inhibitors, ATRT-SHH showed a more heterogeneous response with a subset showing high sensitivity to NOTCH inhibitors, which corresponded to high expression of NOTCH receptors. Our ATRT tumoroids represent the first pediatric brain tumor organoid model, providing a representative pre-clinical model which enables the development of subgroup-specific therapies.

## Introduction

Malignant rhabdoid tumors (MRTs) are rare, but aggressive pediatric tumors. Genetically, MRTs are characterized by the recurrent loss of *SMARCB1* (95% of the cases) or *SMARCA4* (5% of the cases) [1–3]. They can arise in any body part, but usually occur in the kidney and the brain (so-called atypical teratoid/rhabdoid tumors (ATRTs)) [2]. ATRTs make up ~1–2% of all CNS tumors in children, primarily affecting infants (>70%). Despite multi-modal therapy, consisting of a combination of surgery, radiation, high dose chemotherapy, and/or intra thecal chemotherapy, the reported overall survival of children with ATRT remains dismal [4–7].

A closer examination of the epigenetic landscape of ATRTs showed that three subgroups can be distinguished based on DNA methylation patterns and transcriptome profiles, called MYC, Sonic hedgehog (SHH) and Tyrosinase (TYR) [8–10]. However, despite this molecular subclassification, subgroup-specific treatment protocols have so far not been developed [2, 8], which is primarily caused by the lack of representative pre-clinical models. Previous research has primarily relied on the use of a limited set of established cancer cell lines and patient-derived xenograft orthotopic (PDOX) models [11–14]. Although valuable, most of these models either do not resemble primary tumor tissue or do not recapitulate the different molecular ATRT subgroups [9, 15].

Organoid technology has revolutionized cancer research by allowing the efficient establishment and expansion of 3D cell cultures from healthy and diseased tissues such as tumors [16, 17]. Organoids derived from tumor tissue (tumoroids) were demonstrated to preserve many tumor characteristics over long-term passaging [18, 19]. In addition, they were demonstrated to be predictive for patient-specific drug responses [20–24]. However, tumoroid models of brain tumors have not been widely established and to date there are no tumoroid models that recapitulate the different ATRT subgroups. Here, we describe the establishment of patient-derived tumoroid models from the MYC and SHH ATRT subgroups. We demonstrate that the tumoroids represent the patient tumors’ molecular characteristics and provide a scalable pre-clinical platform to investigate subgroup-specific drug vulnerabilities.

## Results

### Tumoroids can be efficiently established from different ATRT subgroups

**Fig. 1:**
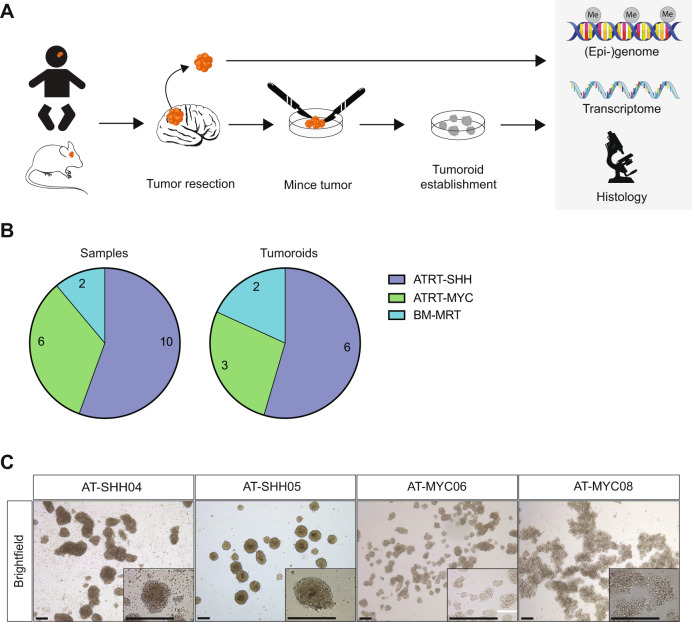
Establishment of ATRT tumoroid models from primary tissues. **A** Schematic overview of the tissue handling workflow and downstream applications. **B** Pie chart representing number of collected samples subdivided per subgroup and paired number of successful tumoroid model establishment. Details of each tumoroid model are provided in Table 1. **C** Brightfield microscopy images of ATRT tumoroid models. Scale bars equal 100 µm.

ATRT is a rare tumor entity and available primary patient material is limited. We therefore set out to develop tumoroid models from both primary patient tumor tissues as well as from PDOX tumor tissues [11] (Fig. 1A). Resected tissue was minced and plated as small tissue fragments in medium optimized for long-term expansion of brain tumor cells. The composition was based on previously described dependencies of ATRT tumor proliferation [10] and other brain tumor cell lines [25] (Fig. 1A). Initially, tissue fragments were embedded in basement membrane extract (BME) and simultaneously grown as suspension culture in the absence of BME. However, in contrast to their extracranial counterparts (extracranial MRTs (ecMRTs)) [19], ATRT tumoroid cultures could typically only be established in the absence of BME, as suspension cultures, which could be reflective of their different micro-environment compared to ecMRTs (Supplementary Fig. S1A). In the presence of BME, tumor cells displayed a differentiation-like phenotype with very limited proliferation (Supplementary Fig. S1A). Three dimensional, multicellular structures were typically observed within 1–2 weeks after initial plating (Supplementary Fig. S1B). On average, ATRT tumoroids were passaged every 7–10 days in a 1:3–1:6 ratio. Established tumoroid cultures can be expanded long term (to date, more than 35 passages), cryopreserved, and recovered successfully upon thawing. Following this protocol, we established six ATRT-SHH (out of ten) and three ATRT-MYC (out of six) tumoroid models. We also established tumoroids of two (out of two) brain metastases of ecMRTs, which we included in all downstream analyses and referred to as brain metastasis MRT (BM-MRT) (Fig. 1B and Table 1). We did not observe a significant difference in establishment efficiency between ATRT-SHH and ATRT-MYC. However, the less aggressive ATRT-TYR subgroup is not represented in our cohort due to limited sample availability. Establishment efficiencies were not influenced by tumor location or prior treatment of the patient (Table 1). Morphologically, ATRT tumoroids appeared as grape-like structures forming either discohesive (e.g., AT-MYC08) or more compact sphere-like structures (e.g., AT-SHH05) (Fig. 1C, Supplementary Fig. S1C). Thus, for the first time, we established culture conditions that allow for the efficient generation of long term expandable tumoroid models of ATRT and brain metastases of ecMRT.

**Table 1:** Clinical characteristics of the tumor samples included in the study.

TUMOROID MODEL	SUBTYPE	SOURCE	GENDER	AGE	TREATMENT	TUMOR LOCATION	SMARCB1 STATUS
AT-SHH02	SHH	PDOX	Female	1 y 3 m	SJYC07 trial	Spinal cord/meninges	Nonsense mutation
AT-SHH04	SHH	Patient	Female	3 y 11 m	EU-RHAB	Frontotemporal lobe	Nonsense mutation
AT-SHH05	SHH	Patient	Male	2 y 4 m	EU-RHAB, MEMMAT	3rd ventricle	Deletion, CN-LOH
AT-SHH15	SHH	Patient	Female	1 m	Naïve	Fossa posterior	Deletion, CN-LOH
AT-SHH17	SHH	PDOX	Female	4 m	SJYC07 trial	Pineal region	Copy number loss
AT-SHH18	SHH	PDOX	Female	5 m	CT, RT, targeted therapy using alisertib	Right parietal lobe	Nonsense mutation
AT-MYC06^a^	MYC	PDOX	Female	4 m	CT, RT, targeted therapy using alisertib	Lung metastasis (from same patient as AT-MYC07)	Copy number loss, nonsense mutation
AT-MYC07^a^	MYC	PDOX	Female	4 m	CT, RT, targeted therapy using alisertib	Lung metastasis (from same patient as AT-MYC06)	Copy number loss, nonsense mutation
AT-MYC08	MYC	PDOX	Female	6 m	CT, RT, targeted therapy using alisertib	Frontotemporal lobe	Copy number loss
BM-MRT01	ecMRT	Metastasis	Female	UNK	UNK	UNK	UNK
BM-MRT20	ecMRT	Metastasis	Male	1 m	Naïve	Cerebellum	Deletion

UNK: unknown (i.e., information not available).

^a^These tumoroid models were derived from independent ATRT metastases from the same patient. Details about treatment protocols are provided in the Material and Methods section.

### ATRT tumoroids retain phenotypic and molecular characteristics of parental tumors

**Fig. 2:**
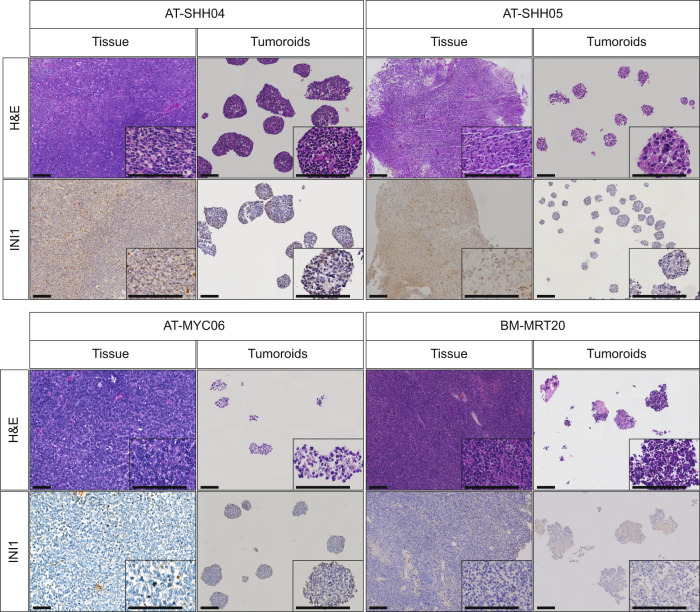
Immunohistochemical characterization of ATRT tumoroid models. H&E and INI1 stainings of the indicated ATRT tumoroid models and matching tissues (H&E = hematoxylin & eosin staining, INI1 = protein encoded by the *SMARCB1* gene). Scale bars equal 100 µm.

Next, we investigated whether the ATRT tumoroids phenotypically resemble the tissues they were derived from. Hematoxylin and eosin (H&E) staining showed that ATRT tumoroids consistently appeared as solid, dense structures composed of cells that, like ATRT tissues, displayed rhabdoid cell features such as abundant eosinophilic cytoplasm, vesicular nuclei, and prominent nucleoli (Fig. 2, Supplementary Fig. S2). As expected, due to the bi-allelic loss of *SMARCB1* in these tumors, INI1 protein expression was absent in ATRT tissues and established tumoroid models (Fig. 2, Supplementary Fig. S1D, Supplementary Fig. S2), confirming in vitro expansion of ATRT cells. Thus, ATRT tumoroids are phenotypically representative of patient tumor tissues.

**Fig. 3:**
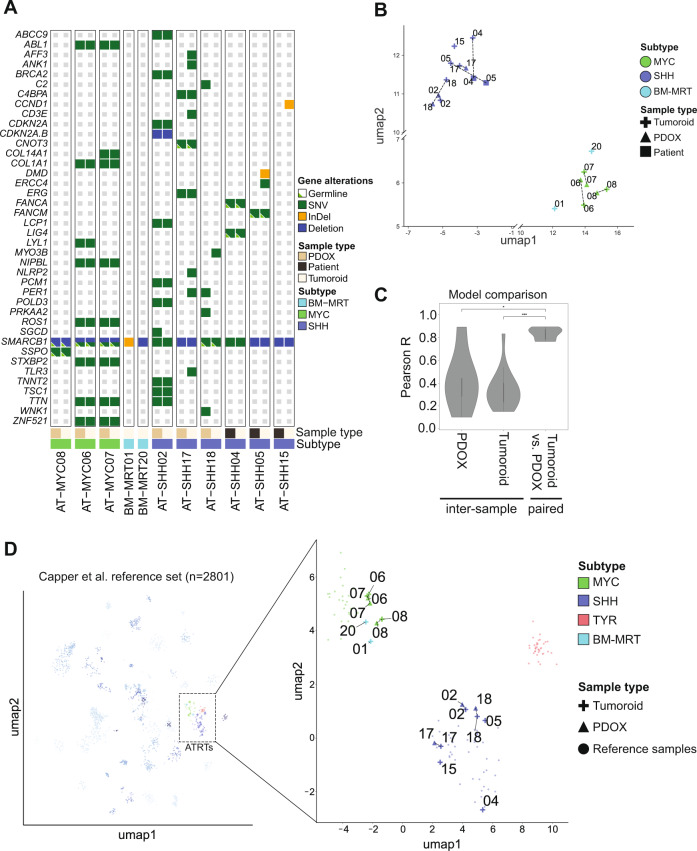
Molecular classification of ATRT tumoroid models. **A** Oncoprint of gene alterations found in ATRT tumoroids and matching parental tissue (patient or PDOX). Germline mutations are indicated by a small green triangle. **B** UMAP plot of paired parental source (primary or PDOX) and tumoroid gene expression profiles generated by RNAseq. Related samples are indicated by a dashed line; subgroup is indicated by color, and sample source indicated by shape. **C** Comparison of sample consistency using the 3000 most variable DNA methylation array probes in the ATRT reference set, applied to the tumoroid and PDOX dataset. The Pearson’s R was calculated for all unique sample comparisons (inter-sample within one subgroup) or paired between two matching sample types (tumoroid versus parental tissue). Model comparisons are performed with the complete dataset, combining MYC inter-sample and SHH inter-sample comparison results (“PDOX”, “Tumoroid”); MYC-paired and SHH-paired comparison results were combined separately (“Tumoroid versus PDOX”). Statistical significance was determined by Mann–Whitney test (*: *p* ≤ 0.05, **: *p* ≤ 0.01, ***: *p* ≤ 0.001). **D** UMAP plot of reference samples, paired parental source (patient or PDOX), and tumoroid DNA methylation profiles. Related samples are indicated by dashed line; subgroup is indicated by color, and sample source indicated by shape.

We then summarized the genetic profiles of ATRT tumoroids and, if available, matching primary patient or PDOX samples by whole genome sequencing (WGS) or whole exome sequencing (WES). To ensure that the tumoroids matched the molecular profiles of their parental source, we focused our analysis on coding genes of known or putative status (Fig. 3A) [26]. Overall, we observed that the molecular alterations of the tumoroid models showed a high degree of similarity when compared to the primary tumor. Of the eleven tumoroid models generated, all showed bi-allelic *SMARCB1* gene alterations (Fig. 3A, Supplementary Table 1). To further confirm that ATRT tumoroids preserve the genetic landscape of patient tumors, we extracted mutational signatures from the WGS/WES data of our tumoroids, as well as WGS/WES data of matching tissue samples. We subsequently compared these to recently described mutational signatures in the Catalogue of Somatic Mutations in Cancer (COSMIC) database. This analysis revealed the presence of signatures SBS01 and SBS05 across all samples, which are commonly found in cancer (Supplementary Fig. S3A) [27]. Furthermore, platinum-based therapy-related signatures SBS31 and SBS35 [28] were detected in several tumor tissues as well as their matching ATRT tumoroid model (Supplementary Fig. S3A). Overall, the mutational signatures found in patient tumor tissues are represented in the ATRT tumoroid models.

A crucial aspect for a representative pre-clinical ATRT model is that it maintains the features defining the different molecular subgroups. To investigate whether our ATRT tumoroid models maintain such subgroup-specific features, we subjected tumoroids and their matching tumor tissues to bulk RNA sequencing (RNAseq). Unsupervised clustering using Uniform Manifold Approximation and Projection (UMAP) [29] showed that tumoroids clustered based on subgroup, with ATRT-SHH and ATRT-MYC tumoroids clustering with their respective primary sample (Fig. 3B, Supplementary Fig. S3B). BM-MRT tumoroids clustered together with the ATRT-MYC subgroup (Fig. 3B, Supplementary Fig. S3B), which is consistent with recent reports indicating that ecMRTs primarily resemble ATRT-MYC [10, 30, 31]. Using published marker gene sets for ATRT-MYC and ATRT-SHH subgroups [9], we confirmed strong specificity of marker gene expression of the ATRT tumoroid model with the corresponding subgroup hallmark gene set (Supplementary Fig. S3C).

To further verify that epigenetic-based subgroup signatures are maintained in culture, we analyzed DNA methylation profiles of the ATRT tumoroid models in comparison to their parental source (parental data only available for PDOX models). Using the Pearson correlation as a measure of consistency between samples, the top 3000 most variably methylated probes were compared across the different sample models (Fig. 3C, Supplementary Tables 2 and 3). Pearson correlation of matching PDOX-tumoroid pairs was significantly higher than correlation scores within the PDOX or tumoroid samples (Fig. 3C, Supplementary Fig. S3D and S4A, B, Supplementary Table 2) (inter-sample PDOX versus paired model per subgroup *p* = 0.04533; inter-sample tumoroid versus paired model *p* = 5.962e−04). Furthermore, we classified the methylation profiles of the ATRT tumoroid models with a previously published method trained on a wide range of pediatric brain tumor DNA methylation profiles [32] (Supplementary Table 4). Unsupervised clustering against this cohort revealed that ATRT tumoroids exclusively clustered with the subgroup they were derived from (Fig. 3D, Supplementary Fig. S3D and S4A, B, Supplementary Table 4).

Next, we investigated whether the ATRT tumoroid models preserve the genetic and epigenetic features over long-term passaging. To do so, we performed WGS and DNA methylation analyses on four late passage (>passage 30) tumoroid models. We did not observe any large chromosomal aberrations in any of the late passage cultures (Supplementary Fig. S5). Furthermore, comparing VAFs of called SNVs and insertion/deletions (indels) in coding regions of early and matched late passage cultures revealed that the majority of variants are retained. Therefore, genetic heterogeneity is largely maintained during long-term passaging (Supplementary Fig. S6). We observed 6 to 20 additional SNVs in coding regions of late passage tumoroid models compared to the matched early passage models (Supplementary Fig. S4C, D). The vast majority involved missense mutations and none of the mutations occur in known cancer-driving genes. (Supplementary Fig. S4C, E). Finally, DNA methylation analysis revealed consistent clustering of the primary tissue sample with both the early and late passage tumoroid samples (Supplementary Fig. S4F). Altogether, our analyses confirm that ATRT tumoroids retain the genetic features of ATRT tumors as well as ATRT subgroup-specific DNA methylation and gene expression signatures.

### Drug screening on ATRT tumoroids reveals subgroup-specific therapeutic vulnerabilities

**Fig. 4:**
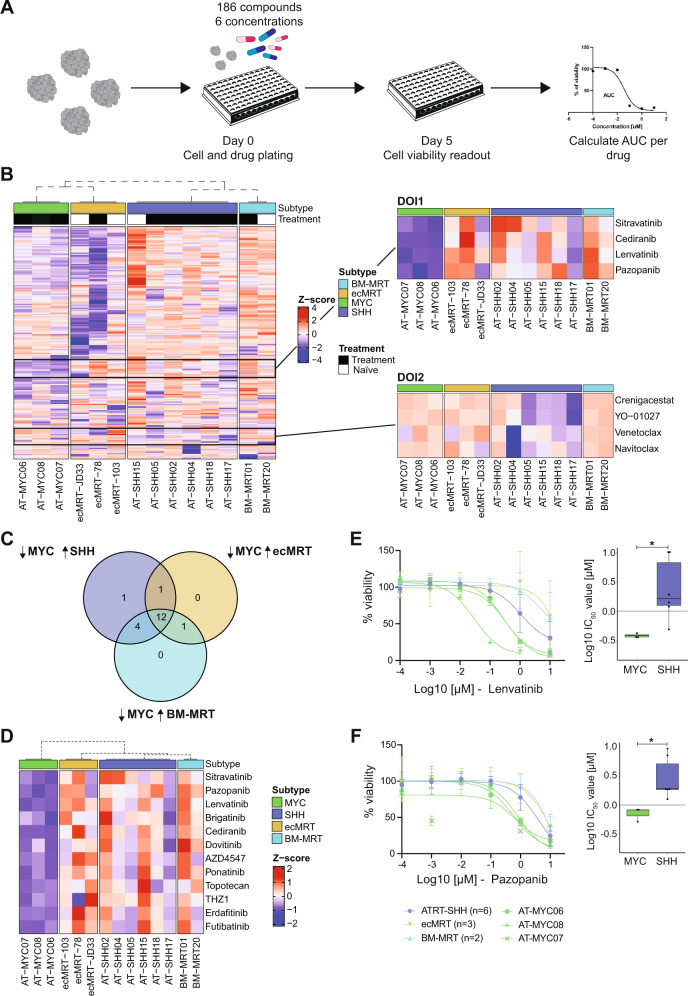
High throughput drug screens reveal subgroup-specific vulnerabilities. **A** Schematic overview of the drug screen workflow. **B** Clustering based on subgroup using *z*-scores calculated per row for 186 drugs. Black boxes indicate drugs of interest (DOI) regions within the left heatmap. DOI1 depicts the four top hits for the ATRT-MYC subgroup. DOI2 depicts the four top hits for the ATRT-SHH subgroup. **C** Identification of ATRT-MYC-specific compounds by comparing ATRT-MYC (*z*-score < −1) versus the other tumor entities (*z*-score > 0). **D** Heatmap of the *z*-scores of the 12 drugs of interest. Dashed dendrogram indicates supervised clustering by subgroup. **E** Dose response curve of Lenvatinib (left) and corresponding Log10 IC_50_ values per subgroup (right) (two-tailed unpaired Wilcoxon test; *: *p* ≤ 0.05). **F** Dose response curve of Pazopanib (left) and corresponding Log10 IC_50_ values per subgroup (right) (two-tailed unpaired Wilcoxon test; *: *p* ≤ 0.05).

Although recent studies suggest that the different ATRT molecular subgroups have distinct clinical features [33], subgroup-specific treatment regimens have thus far not been developed. To investigate whether ATRT tumoroid models will allow for identification of novel drug vulnerabilities and therapies, we performed high throughput drug screens on our established ATRT, BM-MRT, and ecMRT [34] tumoroids. We included ATRT-SHH (*n* = 6), ATRT-MYC (*n* = 3), BM-MRT (*n* = 2), and ecMRT (*n* = 3) tumoroid models and screened a drug library developed in-house, containing 186 drugs (Fig. 4A, Supplementary Table 5). To investigate subgroup-specific differences in drug response, dose response curves were generated and *z*-scores of area under the curve (AUC) values were calculated for each tumoroid model. Drugs of interest (DOI) were identified as MYC-specific (DOI1) or SHH-specific (DOI2) by *z*-score comparisons (Fig. 4B). To exclude that the observed effects were caused by doubling times, we correlated AUC values with the corresponding proliferation rates for each model and found no positive, or negative correlations (Supplementary Fig. S7A).

To identify ATRT-MYC specific drug sensitivities, we selected drugs with a *z*-score lower than −1 exclusively for ATRT-MYC tumoroids (Fig. 4C, Supplementary Fig. S7B). In total, 12 drugs were identified selectively targeting ATRT-MYC and none of the other subgroups (Fig. 4C). Strikingly, ten of these were multiple tyrosine kinase inhibitors (mTKIs) (Fig. 4D, Supplementary Fig. S7C). The other two identified drugs were the topoisomerase 1 inhibitor Topotecan and the covalent CDK7 (THZ1) inhibitor which, upon AUC value inspection, showed a high potency in each subgroup (Supplementary Fig. S7D, Supplementary Table 6). The mTKIs Lenvatinib and Pazopanib showed the most significant difference between the ATRT-MYC and ATRT-SHH subgroups (Fig. 4E; Lenvatinib *p* = 0.028, Fig. 4F; Pazopanib *p* = 0.024). ATRT tumoroid models are cultured in medium supplemented with EGF, FGF2, PDGF-AA and PDGF-BB. To exclude that the increased sensitivity of ATRT-MYC models to mTKIs is culture induced, we depleted one or a combination of growth factors from the cell culture medium. Cell viability was assessed using CellTiterGlo and Annexin V apoptosis assays. We observed that proliferation and viability of ATRT-MYC and ATRT-SHH tumoroid models were equally dependent on the presence of FGF2 in the culture medium (Supplementary Fig. S8A–D). In contrast, both ATRT-MYC and ATRT-SHH cultures were not significantly affected by the depletion of PDGF-AA, PDGF-BB, or EGF, indicating that ATRT-MYC and ATRT-SHH tumoroid models are dependent on the same growth factors. Therefore, the increased sensitivity of ATRT-MYC tumoroids to mTKIs is not induced in vitro. To identify whether the ten identified mTKIs inhibit a common target, we performed k-means clustering on z-scores for all forty-eight mTKIs included in the drug library. We found a cluster including the ten mTKIs that selectively suppressed proliferation of all ATRT-MYC tumoroid models (Supplementary Fig. S9A). Notably, this cluster was not defined by targeting one shared kinase (Supplementary Fig. S9A). Specificity of these mTKIs toward ATRT-MYC tumoroid models was further investigated by comparing their drug sensitivity to the sensitivity of several other pediatric tumor entities [18, 19]. Consistently, ATRT-MYC tumoroids showed a significantly lower AUC value for Pazopanib and Lenvatinib compared to all other tumor entities (Supplementary Fig. S9B, C).

To explore which kinases were influenced by our DOIs, we analyzed the phosphorylation status of a panel of 49 different receptor tyrosine kinases (RTKs) in the presence and absence of the mTKI Lenvatinib for the tumoroid models AT-MYC07 (sensitive) and AT-SHH15 (resistant). Comparing the non-treated samples, we detected phosphorylation of FGFR2α, PDGFRα, and the insulin receptor in AT-MYC07 (Supplementary Fig. S9D, E). In contrast, phosphorylation of these RTKs was below detection levels in AT-SHH15 (Supplementary Fig. S9E). Notably, AT-SHH15 did not display phosphorylation of any of the 49 RTKs. Upon Lenvatinib treatment, a strong reduction in phosphorylated FGFR2α and PDGFRα, but not the insulin receptor was observed in AT-MYC07 (Supplementary Fig. S9D, E). Taken together, ATRT-MYC tumoroid models show high basal phosphorylation levels of the receptor tyrosine kinases PDGFRα and FGFR2α that are selectively downregulated upon mTKI treatment, possibly explaining the increased vulnerability of ATRT-MYC to mTKIs.

**Fig. 5:**
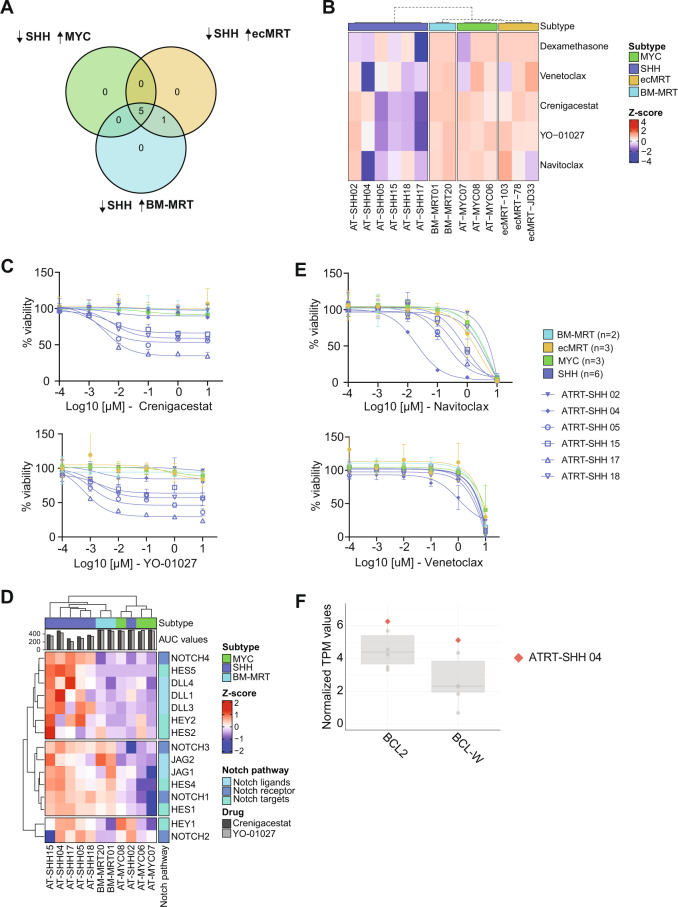
Patient-specific drug vulnerabilities within the ATRT-SHH subgroup. **A** Identification of ATRT-SHH-specific compounds by comparing SHH (*z*-score < −0.5) versus the other tumor entities (*z*-score > 0). **B** Heatmap depicting *z*-scores of the five drugs of interest for the ATRT-SHH subgroup. Dashed dendrogram indicates supervised clustering by subgroup. **C** Dose response curves of Crenigacestat and YO-01027 (gamma-secretase inhibitors). Sample subgroups are indicated by color and individual SHH samples are indicated by shape and plotted individually. **D** Heatmap showing *z*-scores of trimmed mean of M values (TMM) normalized gene expression for all ATRT tumoroid models for several genes in the NOTCH pathway. **E** Dose response curves of Navitoclax and Venetoclax. Sample subgroups are indicated by color and individual SHH samples are indicated by shape and plotted individually. **F** Representation of *BCL2 and BCL-W* gene expression in normalized TPM values of ATRT-SHH tumoroid models. AT-SHH04 is highlighted as the only responder of the ATRT-SHH tumoroid model (red square).

We identified five drugs (Fig. 5A, Supplementary Fig. S10A) having a heterogenous response in the ATRT-SHH tumoroid models (Fig. 5B, Supplementary Fig. S10B). Four out of the five ATRT-SHH tumoroid models showed an increased sensitivity toward the gamma-secretase inhibitors Crenigacestat and YO-01027 (Fig. 5C). As gamma-secretase inhibitors specifically inhibit NOTCH signaling, we analyzed gene expression levels of hallmark genes within the NOTCH pathway [35, 36]. Compared to BM-MRT and ATRT-MYC tumoroids, expression levels of NOTCH receptors, ligands, and main targets were markedly higher in five out of six ATRT-SHH tumoroid models (Fig. 5D). In contrast, the most resistant tumoroid model, AT-SHH02, did not display increased expression of these NOTCH pathway components, possibly explaining its decreased sensitivity toward gamma-secretase inhibitors (Fig. 5D). Furthermore, AT-SHH04 showed increased sensitivity to the BCL-2 inhibitors Navitoclax and Venetoclax compared to the other ATRT-SHH models (Fig. 5E). Interestingly, this model displayed high expression of *BCL-2* and *BCL-W* (Fig. 5F). Taken together, we demonstrate that ATRT tumoroids can be used as a pre-clinical platform for high-throughput drug screens, allowing for the identification of subgroup-specific and patient-specific drug vulnerabilities.

## Discussion

The advances in three-dimensional cell culture systems, such as organoids, has innovated cancer research and the development of individualized therapy [16]. Especially for rare tumor entities, such as ATRTs, patient-derived tumor organoid (tumoroid) cultures can be of great value to fill the void of representative pre-clinical models. In this study, we describe the establishment of the first long term expandable tumoroid model for a pediatric brain tumor, ATRT. We demonstrate that tumoroid models can be efficiently generated from ATRT-SHH and ATRT-MYC subgroups preserving molecular and phenotypic characteristics of their parental tumor with little divergence upon long-term culturing. As cycling cells are known to accumulate mutations over time [37, 38], genetic diversification of the culture is inevitable and an inherent feature of cycling cells [37, 38]. Whether such genetic diversification reflects tumor evolution in patients remains an outstanding question. So far, we have not been able to establish tumoroid cultures of the ATRT-TYR subgroup. Notably, efforts to grow ATRT-TYR tumors in vivo for more than one passage have also failed. To our knowledge no ATRT-TYR PDOX models have been generated so far. Several studies demonstrated ATRT-TYR tumors to be less aggressive than ATRT-SHH and ATRT-MYC [6, 7], which could explain why it is more challenging to grow them in vitro and in vivo.

Our ATRT tumoroids serve as representative models to explore subgroup-specific tumorigenesis and to identify novel therapeutic targets. We identified a high sensitivity of ATRT-MYC tumoroids to a subgroup of mTKIs. Indeed, we found that compared to ATRT-SHH tumoroids, ATRT-MYC tumoroids have high basal levels of phosphorylated FGFR2α and PDGFRα. The observed decrease in the phosphorylation of these receptors upon mTKI treatment as well as dependency on FGF2 for cell proliferation suggests that the activity of these two receptors at least partially underpins the increased vulnerability of ATRT-MYC tumors to mTKI treatment. These results are in line with the findings of Torchia and colleagues who previously identified ATRT sensitivity toward Dasatinib and Nilotinib [10]. The number of ATRT-MYC tumoroid models we have established so far is still small and extrapolating our findings to a larger set of ATRT-MYC models will be needed to further reinforce our observations. Interestingly, although ecMRTs are known to primarily resemble the ATRT-MYC subgroup on the epigenetic level [31], ecMRTs and BM-MRTs did not display high sensitivity to mTKIs. In fact, ecMRT and BM-MRT tumoroids displayed distinct responses in the drug screen, which could be caused by their different cellular origins. Exploring the differences between MYC-driven MRTs in more detail may ultimately lead to patient-tailored treatment regimens.

While we observed consistent drug sensitivity across all screened ATRT-MYC models, ATRT-SHH tumoroid models displayed strong intertumoral differences in drug response. This could be explained by the recent observation that the ATRT-SHH subgroup can be further subdivided into three subgroups with different clinical outcomes [39]. Drug testing on larger collections of ATRT-SHH tumoroid models will be required to determine whether the observed differences in drug sensitivity are indeed caused by subgroup differences. Moving forward, extensive in vivo studies using ATRT PDOX models as well as investigations into the development of combination treatments will be required to translate our findings to the clinic.

## Material and methods

### Ethics statement

The study was approved (MEC-2016-739) by the medical ethical committee of the Erasmus Medical Center (Rotterdam, the Netherlands) and Princess Máxima Center for Pediatric Oncology (Utrecht, The Netherlands). For PDOX generation, consent to collect patient samples for development of PDOX was reviewed by the St. Jude Children’s Research Hospital Institutional IRB and implemented under the protocol NBTP01. Informed written consent was provided by all patients and/or guardians.

### Treatment protocols of patient samples included in this study

Patients’ treatment differed depending on age and country of treatment from treatment-naïve, standard EU-RHAB [6] and MEMMAT protocol [40] to SJYC07 trial [7] or a combination of chemo-, radio-, and targeted therapy [41] (Table 1).

### Patient-derived orthotopic xenografts

ATRT patient-derived orthotopic xenografts (PDOXs) were previously described [11].

### Primary tissue processing

Following resection, a viable piece of tumor was selected from the resected material. One or two small representative pieces were cut and fixed in formalin for downstream histopathological analysis. The rest of the tumor pieces were minced into 1–3 mm^3^ pieces, rinsed with ice-cold medium (DMEM 1X GlutaMax with 4,5 g/L D-Glucose, Pyruvate, and PenStrep; ThermoFisher) and centrifuged at 250 × *G* at 4 *°*C. If a red pellet was visible, 2–3 mL red blood cell lysis buffer (Roche) was added and cells were incubated for 5 min at room temperature. Washing step was repeated and cells were taken in culture and cryopreserved.

### Tumoroid cultures

Cells were seeded in tumor stem medium (TSM) containing a 50:50 mix of DMEM:F12 (ThermoFisher) and Neurobasal medium A (ThermoFisher) supplemented with B27 supplement without Vitamin A (Gibco), N2 (Gibco), Heparin (5 IU/ml, ThermoFisher), epidermal growth factor (EGF; 31.25 ng/mL, Peprotech), fibroblast growth factor (FGF2; 31.25 ng/mL, Peprotech), platelet derived growth factor AA (PDGF-AA; 10 ng/mL, Peprotech), and PDGF-BB (10 ng/mL, Peprotech). Medium was refreshed every 3–4 days and cultures were passaged every 7–10 days in a 1:3–1:6 ratio. For passaging, tumoroids were disrupted using mechanical dissociation. After addition of 5–10 mL ice-cold DMEM, cells were centrifuged at 300 × *G* for 5 min at 4 °C and subsequently replated in fresh TSM. All tumoroid models are stored in the biobank of the Princess Máxima Center or St. Jude Children’s Research Hospital and are available to the scientific community according to the rules and regulations under which the patients and parents gave informed consent for donating the tissue.

### Western Blot analyses

Cells were harvested in RIPA buffer followed by sonication of the lysate. Western blot was performed as previously described [42]. The following primary antibodies were used: anti-INI1 (Santa Cruz, sc166165) and anti-GAPDH (Abcam, ab9485).

### Phospho-RTK array

ATRT tumoroid models (AT-SHH15 and AT-MYC07) were treated for 48 h with 1 µM Lenvatinib or DMSO. Cells were lysed according to manufacturer’s instructions. Total protein levels were measured by BCA assay (ThermoFisher) and 150 µg total protein was subsequently used for the assay. Human phospho-RTK array was performed following the manufacturer’s instructions (R&D systems, ARY001B). Signal quantification was performed using ImageJ (version 2.0.0).

### Growth factor withdrawal experiments

ATRT tumoroids models were cultured for 5 days in TSM lacking either PDGF-AA and PDGF-BB, EGF, FGF2 or all four growth factors. Cell viability was assessed at day 0 and day 5 using CellTiter-Glo 3D reagent (Promega) according to manufacturer’s instructions. Cell death was measured using Annexin V/ DAPI staining as previously described [34].

### Histology and immunohistochemistry

Tissues and tumoroids were fixed in 4% paraformaldehyde, dehydrated, and subsequently embedded in paraffin. Sections were cut (4 µm) and subsequently subjected to hematoxylin and eosin (H&E) or immunohistochemical (IHC) stainings according to standard protocols. The following primary antibody was used: anti-INI-1 (Cellmarque, 760-4615, 1:200).

### Whole genome and whole exome sequencing

Genomic DNA was extracted from tumoroids (early passage between 4 and 15; late passage between 30 and 35) and, if available, matching tumor tissues using the ReliaPrep gDNA Tissue Miniprep System (Promega) according to the manufacturer’s instructions. Each DNA sample was sequenced in a paired-end approach for 150 cycles using the NovaSeq 6000 platform with a 30X base coverage for tumoroids and 90X for tissue. All whole genome (WGS) and whole exome sequencing (WES) analyses were performed as previously described [11]. Both somatic and germline mutations were annotated by Medal Ceremony, and reported as Gold, Silver, Bronze, or Unknown [26]. A minimum of 4 variant-called reads and at least 10× coverage was required support for both somatic and germline variants. Germline mutations were further filtered to include only variants with strong support, including Medal Ceremony Gold annotation, variant allele frequency (VAF) ≥ 0.2, and a population frequency as reported by ExAC <0.001. Somatic variants called from primary tumor, PDOX, and tumoroid samples without paired germline sequencing data were called and retained in the same manner as germline mutations. Only non-synonymous somatic variants were reported, including nonsense, missense, splice mutations, frameshift, in-frame insertion/deletion. Somatic variants annotated as Gold, Silver, or Bronze were retained. All somatic variants with paired germline sequencing data were additionally filtered by the following criteria: (1) minor allele frequency <0.01 in NHLBI, 1000 Genomes, and ExAC databases, and (2) have a minimum VAF of 10%. All germline and somatic variants which passed their respective filtering criteria were manually confirmed by examining the mapped reads. All somatic variants which passed these thresholds were manually re-examined in other matching samples in which they were not originally called. Venn diagrams depicting non-synonymous mutations in early and late passaged tumoroids follow the same criteria as above, without filtering by Medal Ceremony annotations. All CNVs were called by CONSERTING [43]. All Gold, Silver, and Bronze annotated somatic CNVs were retained and manually examined; germline CNVs and CNVs in samples without paired germline sequencing data were confirmed in Gold annotated genes by manually examining mapped read coverage. Mutational signature analysis was performed using SigProfiler [44] for the nine tumoroid samples with paired germline WGS or WES data.

### DNA methylation array analysis

DNA of tumoroids and, if available, matching tumor tissues were analyzed using the Illumina Infinium Methylation EPIC array according to the manufacturer’s protocol (Illumina). The MNP pipeline for brain tumor classification and CNV analysis was used (www.molecularneuropathology.org, version 12.5) [32]. For all samples, beta values were calculated using the R package minfi (v1.28.4) [45] with ssNoob normalization [46]. All tumoroid and PDOX samples passed QC with >98% probe coverage and >1 bisulfite conversion ratio. Autosomal probes were retained; probes which did not pass initial QC (*p* < 0.05 using the minfi function detectionP) in at least 10% of samples of the same class were removed from downstream analyses. After combining references samples [32] and EPIC array datasets, 413 323 probes remained. Unsupervised clustering was performed using UMAP v0.5.1 dimensionality reduction implemented in python v3.7.9 [29] of the top 3000 most variable probes as determined by median absolute deviation (MAD). DNA methylation sample consistency was performed by calculating Pearson’s R across inter-sample and paired-sample comparisons, using the top 3000 most variable probes as determined by MAD in the ATRT reference set. Differentially methylated probes (from the top 3000 most variable probes, as plotted in the ATRT-only UMAP) were called using the R package limma (v3.52.2) [47] (adj.P.val < 0.05, logFC > 2). Subsequent gene set analyses were performed using R package missMethyl (1.30.0) [48]. The differentially methylated region (DMR) analysis was performed using all autosomal probes passing QC criteria as described above, with R package DMRcate (v 2.10.0) [49]. Subsequent, gene set analyses were performed with missMethyl function goregion. A copy number variation (CNV) analysis using 850 K EPIC DNA methylation array data was performed with the Conumee Bioconductor package [50].

### Bulk RNA sequencing

Total RNA was extracted from tumoroid samples and, if available, matching tumor tissues using Trizol reagent (Invitrogen). RNA quality and quantity were assessed using Bioanalyzer2100 RNA Nano 6000 chips (Agilent) and Qubit measurement. Total RNA libraries were prepared using the NEBNext Ultra RNA Library Prep Kit (New England Biolabs) by Novogene (UK). Each RNA sample was sequenced using a paired-end approach with the Illumina NovaSeq 6000 platform either at Novogene (UK) (PE150) or St. Jude Children’s Research Hospital (USA) (PE100). For PDOX, sequencing reads were cleaned of potentially contaminating mouse reads using XenoCP [51]. All sample reads were mapped to GENCODE GRCh38 primary assembly release 31 using STAR (v2.7.1) [52] and quantified using RSEM (v1.3.1) [53] using gene definitions from GENCODE release 31. Packages edgeR (v3.24.3) [54] and limma (v3.83.3) [47] in the R environment (v3.5.1) was used to normalize gene expression. A threshold of FDR < 0.05 was used to determine statistical significance for all analyses. Unsupervised hierarchical clustering was visualized using the R package ComplexHeatmap (v2.2.0) [55] with default values. Other PDOX versus tumoroid expression comparisons were tested for statistical significance using the python package scipy (v1.6.0) implementation of Mann–Whitney U test, adjusted for FDR using fdrcorrection (statsmodels, v0.12.2).

### High throughput drug screens

Tumoroids were collected and 500 extra-cranial MRT (ecMRT) tumoroid cells or 3000 ATRT tumoroid cells (all between passage 10 and 30) were plated in a volume of 40 µl in black 384-well plates (Corning) using the Multi-drop Combi Reagent Dispenser (ThermoFisher). The drug screen was performed with a library containing 186 drugs using the high-throughput screening facility at the Princess Maxima Center (Beckman Coulter with a Biomek i7 Automated Workstation) (Supplementary Table 5). Using the Echo 550 dispenser, 100 nL of the drugs (in DMSO or milliQ, at different concentrations) was added to the cells, to yield final concentrations of 0.1 nM, 1 nM, 10 nM, 100 nM, 1 µM and 10 µM (0.25% DMSO or MQ). Several drugs were tested at additional lower concentrations (up to 10 pM) or higher concentrations (up to 200 µM). Cells treated with only DMSO were used as negative controls, whereas cells treated with Staurosporine (final concentration of 10 µM) were used as positive controls. The screen was performed in duplicate per tumoroid model with four technical replicates for each experiment. ATP levels were measured 120 h after addition of the drugs using CellTiter-Glo 3D reagent (Promega) according to manufacturer’s instructions. Results were normalized to DMSO (100% viability) and Staurosporine (0% viability). Area under the curve (AUC) values were calculated by determining the definite integral of the curve [56]. IC_50_ values were calculated by determining the concentrations of the drug needed to achieve a 50% reduction in cell viability (Supplementary Table 6).

To identify subgroup-specific drugs, average *Z*-scores (based on AUC values) of each subgroup were compared against each other. In addition, AUC values were checked manually to filter out false-positive hits. For the ATRT-MYC subgroup, a compound was called as specific hit with a *z*-score below −1 and a *z*-score above 0 for the other subgroups. An ATRT-SHH-specific hit was called with a *z*-score below −0.5 and above 0 for the other subgroups. Supervised group clustering was visualized using the R package ComplexHeatmap (v2.2.0) [55] with default values.

## Supplementary information

Supplementary Figures

Sup. Table 5

Sup. Table 6

Sup. Table 1

Sup. Table 2

Sup. Table 3

Sup. Table 4

## Data Availability

Sequencing data generated in this study has been deposited to EGA (EGAS00001006422, EGAS00001006866, EGAS00001006865, EGAS00001006881).
