# Supplementary material for: Atypical teratoid/rhabdoid tumoroids reveal subgroup-specific drug vulnerabilities
Source: Oncogene. 2023 Apr 5;42(20):1661–71. doi: 10.1038/s41388-023-02681-y (PMC10181938; doi:10.1038/s41388-023-02681-y)
Supplement: Supplementary file 1 — Supplementary Figures [file 41388_2023_2681_MOESM1_ESM.pdf]

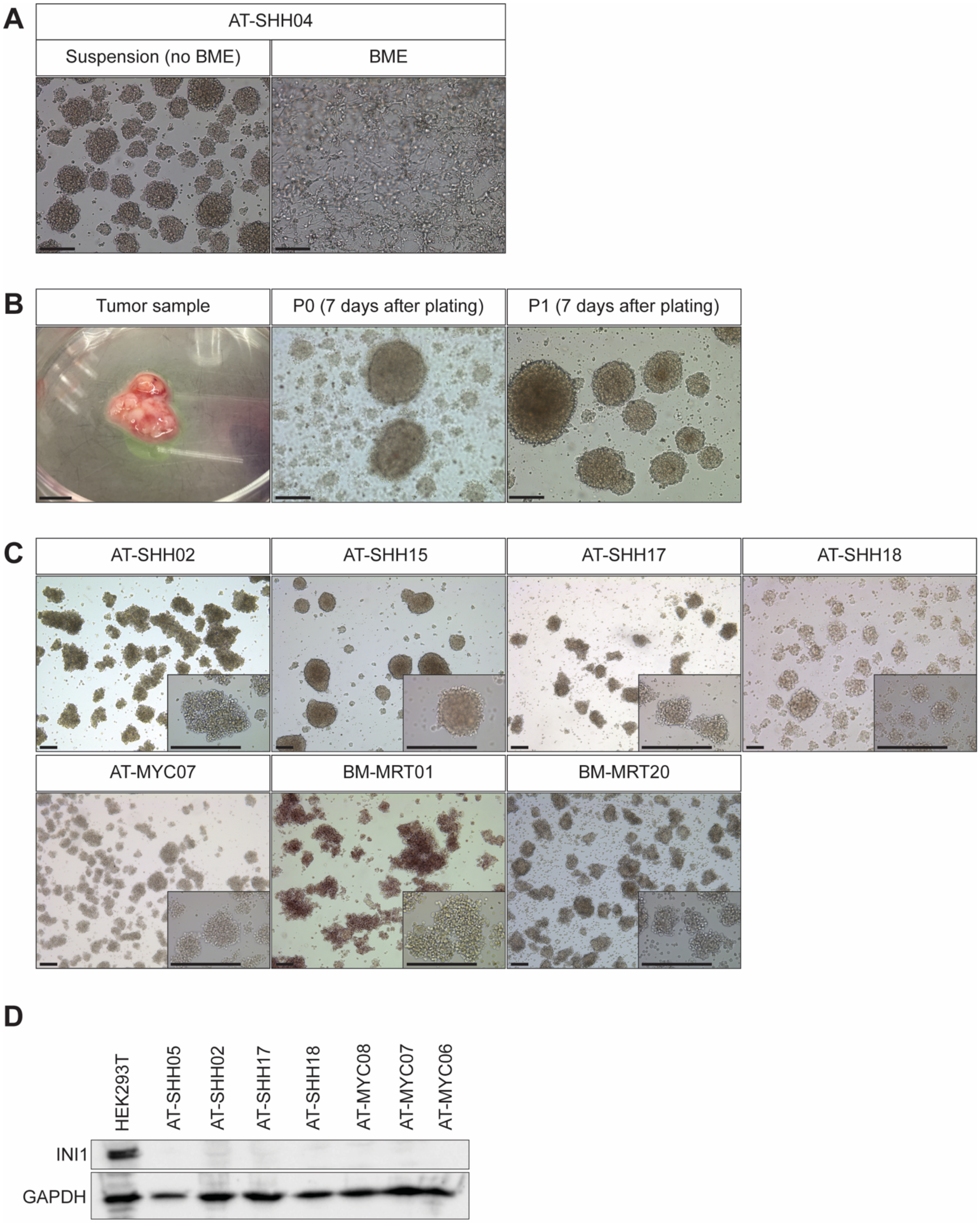
A
AT-SHH04
Suspension (no BME)
BME
B
Tumor sample
P0 (7 days after plating)
P1 (7 days after plating)
C
AT-SHH02
AT-SHH15
AT-SHH17
AT-SHH18
AT-MYC07
BM-MRT01
BM-MRT20
D
HEK293T
AT-SHH05
AT-SHH02
AT-SHH17
AT-SHH18
AT-MYC08
AT-MYC07
AT-MYC06
INI1
GAPDH

Supplementary Figure S1

A Comparison of ATRT tumoroid models cultured in suspension (no BME) or in BME droplets 4 days after seeding. **B** Representative images of tissue processing and plating procedure. Left panel shows starting tumor material (scale bar is 1 cm). Middle panel depicts tumoroid culture 7 days after seeding (P0) and the right panel shows an established tumoroid culture 7 days after seeding (P1). **C** Brightfield microscopy images of established ATRT tumoroid models. Scale bars equal 100 µm (except of S1B left panel, 1 cm). **D** Western blot analyses of INI1 and GAPDH expression in the indicated ATRT tumoroid models and HEK293T cells (positive control).

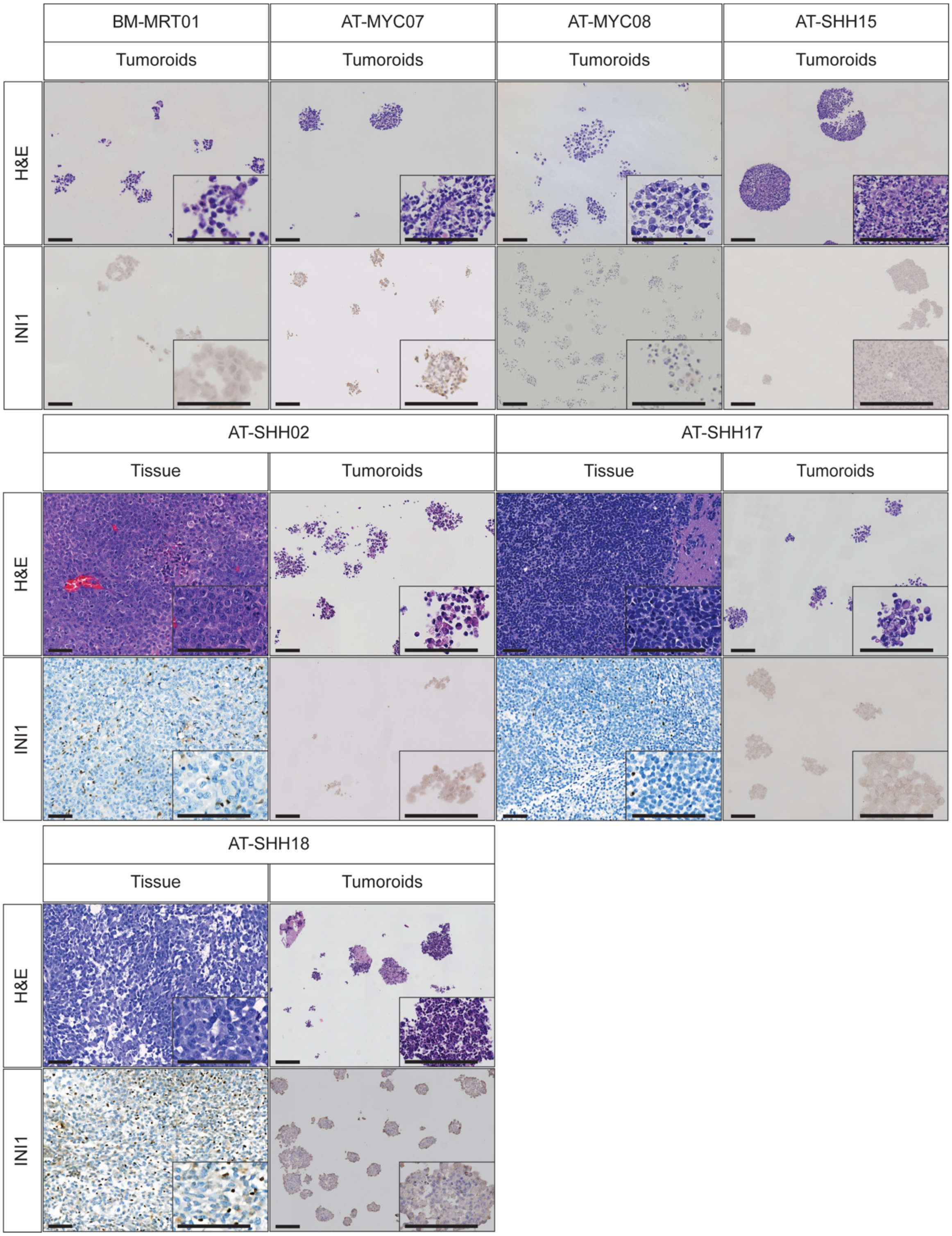
BM-MRT01
AT-MYC07
AT-MYC08
AT-SHH15
Tumoroids
Tumoroids
Tumoroids
Tumoroids
H&E
INI1
AT-SHH02
AT-SHH17
Tissue
Tumoroids
Tissue
Tumoroids
H&E
INI1
AT-SHH18
Tissue
Tumoroids
H&E
INI1

Supplementary Figure S2

Immunohistochemical characterization of ATRT tumoroid models and ,if available, matching tumor tissue (H&E = hematoxylin & eosin staining, INI1 = Protein name of *SMARCB1*). Scale bars equal 100 µm.

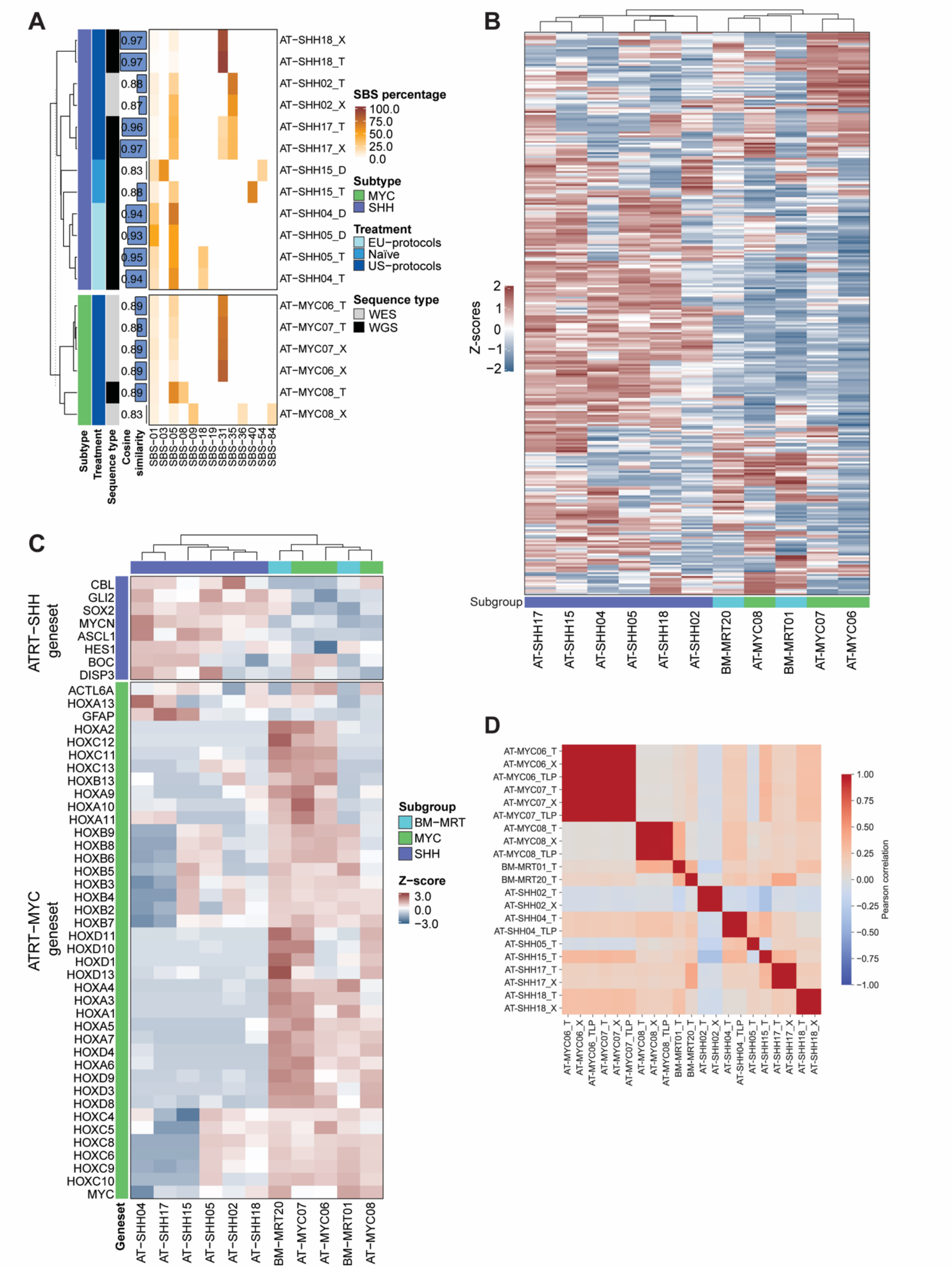
A
AT−SHH18_X
AT−SHH18_T
AT−SHH02_T
AT−SHH02_X
AT−SHH17_T
AT−SHH17_X
AT−SHH15_D
AT−SHH15_T
AT−SHH04_D
AT−SHH05_D
AT−SHH05_T
AT−SHH04_T
AT−MYC06_T
AT−MYC07_T
AT−MYC07_X
AT−MYC06_X
AT−MYC08_T
AT−MYC08_X
0.97
0.97
0.88
0.87
0.96
0.97
0.83
0.88
0.94
0.93
0.95
0.94
0.89
0.88
0.89
0.89
0.89
0.83
SBS percentage
100.0
75.0
50.0
25.0
0.0
Subtype
MYC
SHH
Treatment
EU−protocols
Naïve
US−protocols
Sequence type
WES
WGS
Subtype
Treatment
Sequence type
Cosine similarity
SBS−01
SBS−03
SBS−05
SBS−08
SBS−09
SBS−18
SBS−19
SBS−31
SBS−35
SBS−36
SBS−40
SBS−54
SBS−84
B
Z-scores
2
1
0
−1
−2
Subgroup
AT-SHH17
AT-SHH15
AT-SHH04
AT-SHH05
AT-SHH18
AT-SHH02
BM-MRT20
AT-MYC08
BM-MRT01
AT-MYC07
AT-MYC06
C
ATRT−SHH geneset
CBL
GLI2
SOX2
MYCN
ASCL1
HES1
BOC
DISP3
ATRT−MYC geneset
ACTL6A
HOXA13
GFAP
HOXA2
HOXC12
HOXC11
HOXC13
HOXB13
HOXA9
HOXA10
HOXA11
HOXB9
HOXB8
HOXB6
HOXB5
HOXB3
HOXB4
HOXB2
HOXB7
HOXD11
HOXD10
HOXD1
HOXD13
HOXA4
HOXA3
HOXA1
HOXA5
HOXA7
HOXD4
HOXA6
HOXD9
HOXD3
HOXD8
HOXC4
HOXC5
HOXC8
HOXC6
HOXC9
HOXC10
MYC
Geneset
AT−SHH04
AT−SHH17
AT−SHH15
AT−SHH05
AT−SHH02
AT−SHH18
BM−MRT20
AT−MYC07
AT−MYC06
BM−MRT01
AT−MYC08
Subgroup
BM−MRT
MYC
SHH
Z−score
3.0
0.0
−3.0
D
AT-MYC06_T
AT-MYC06_X
AT-MYC06_TLP
AT-MYC07_T
AT-MYC07_X
AT-MYC07_TLP
AT-MYC08_T
AT-MYC08_X
AT-MYC08_TLP
BM-MRT01_T
BM-MRT20_T
AT-SHH02_T
AT-SHH02_X
AT-SHH04_T
AT-SHH04_TLP
AT-SHH05_T
AT-SHH15_T
AT-SHH17_T
AT-SHH17_X
AT-SHH18_T
AT-SHH18_X
1.00
0.75
0.50
0.25
0.00
−0.25
−0.50
−0.75
−1.00
Pearson correlation

Supplementary Figure S3

Unsupervised clustering and QC analysis of the tumoroid cohort. **A** COSMIC mutational signature analysis of ATRT tumoroids and matching parental tissue. Support for each single base substitution (SBS) signature called is represented by cosine similarity, as calculated by SigProfiler. Hierarchical clustering of SBS signatures was performed for samples by subgroup (T = tumoroid; X = xenograft; D = patient sample; TLP = tumoroid late passage). **B** Unsupervised hierarchical clustering of the topmost 300 variably expressed genes (as determined by median absolute deviation) among tumoroid samples shows a subgroup-specific clustering pattern. Z-scores are calculated by gene across the samples of TMM normalized gene expression. **C** Unsupervised hierarchical clustering of hallmark gene set for ATRT-MYC and ATRT-SHH of all ATRT tumoroid models. Z-scores calculated as stated in B. **D** Heatmap of Pearson's R from SNP DNA methylation array probes (T = tumoroid; X = xenograft; D = patient sample; TLP = tumoroid late passage).

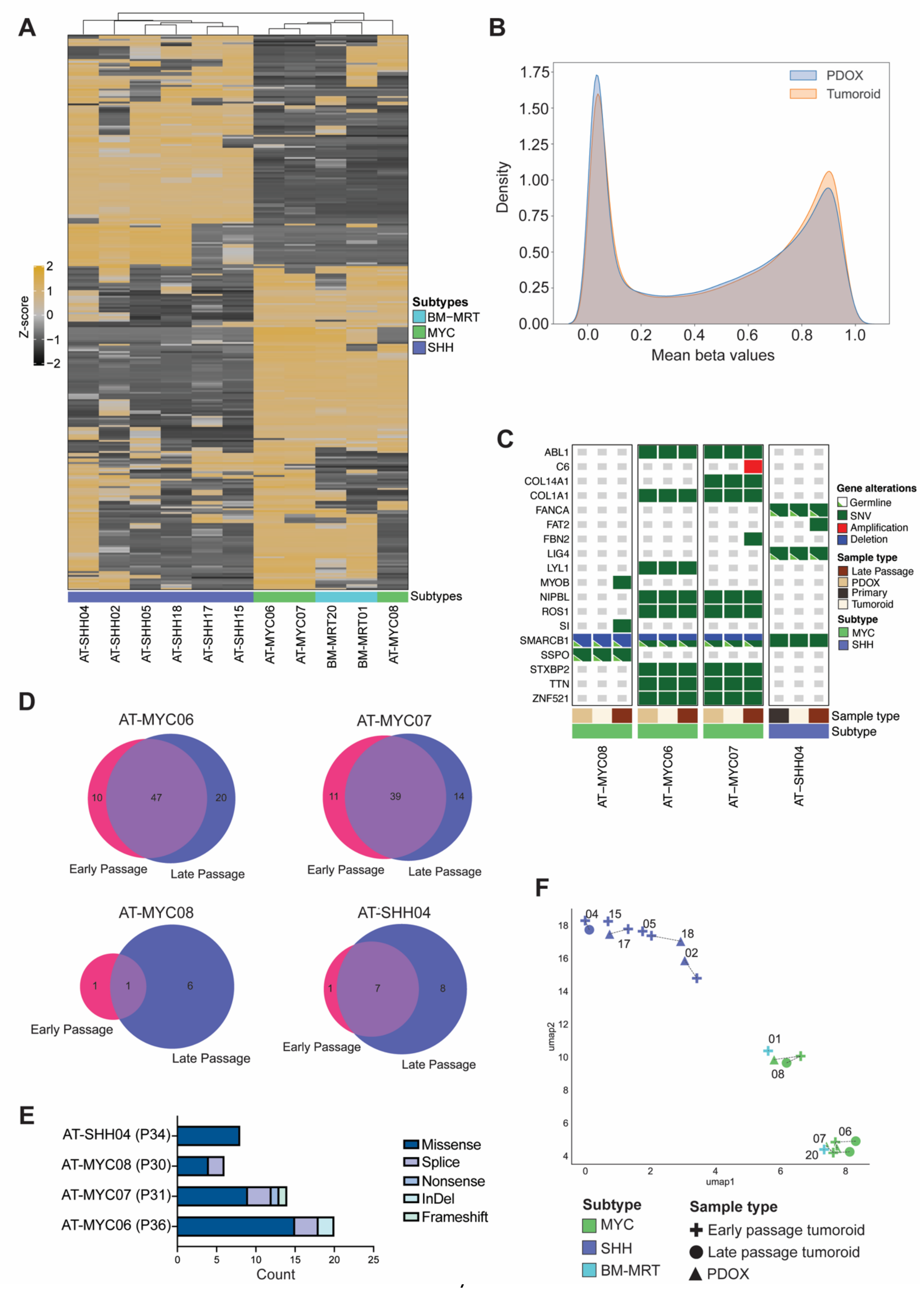
A
Z-score
Subtypes
BM−MRT
MYC
SHH
AT-SHH04
AT-SHH02
AT-SHH05
AT-SHH18
AT-SHH17
AT-SHH15
AT-MYC06
AT-MYC07
BM-MRT20
BM-MRT01
AT-MYC08
B
Density
Mean beta values
PDOX
Tumoroid
C
ABL1
C6
COL14A1
COL1A1
FANCA
FAT2
FBN2
LIG4
LYL1
MYOB
NIPBL
ROS1
SI
SMARCB1
SSPO
STXBP2
TTN
ZNF521
Gene alterations
Germline
SNV
Amplification
Deletion
Sample type
Late Passage
PDOX
Primary
Tumoroid
Subtype
MYC
SHH
Sample type
Subtype
AT−MYC08
AT−MYC06
AT−MYC07
AT−SHH04
D
AT-MYC06
10
47
20
Early Passage
Late Passage
AT-MYC07
11
39
14
Early Passage
Late Passage
AT-MYC08
1
1
6
Early Passage
Late Passage
AT-SHH04
1
7
8
Early Passage
Late Passage
E
AT-SHH04 (P34)
AT-MYC08 (P30)
AT-MYC07 (P31)
AT-MYC06 (P36)
Count
Missense
Splice
Nonsense
InDel
Frameshift
F
umap1
umap2
Subtype
MYC
SHH
BM-MRT
Sample type
Early passage tumoroid
Late passage tumoroid
PDOX

Supplementary Figure S4

A Unsupervised hierarchical clustering of the 300 most variable methylated probes (as determined by median absolute deviation) among tumoroid samples shows clustering based on molecular subgroup. Z-scores are calculated on minfi normalized DNA methylation signal by CpG probe. **B** Comparison of mean beta value DNA methylation distributions between tumoroids and matching parental samples. **C** Oncoprint of gene alterations found in ATRT tumoroids, tumoroids at late passage, and matching parental tissue (patient or PDOX). Germline mutations are indicated by a small green triangle. **D** Venn diagrams showing the overlap between early and late passage tumoroids of all tier 1 somatic mutations (VAF cut-off = 0.1). **E** Summary of class of mutations found in late passage tumoroids. **F** UMAP depicting the top 3,000 most variable methylated probes (as determined by median absolute deviation).

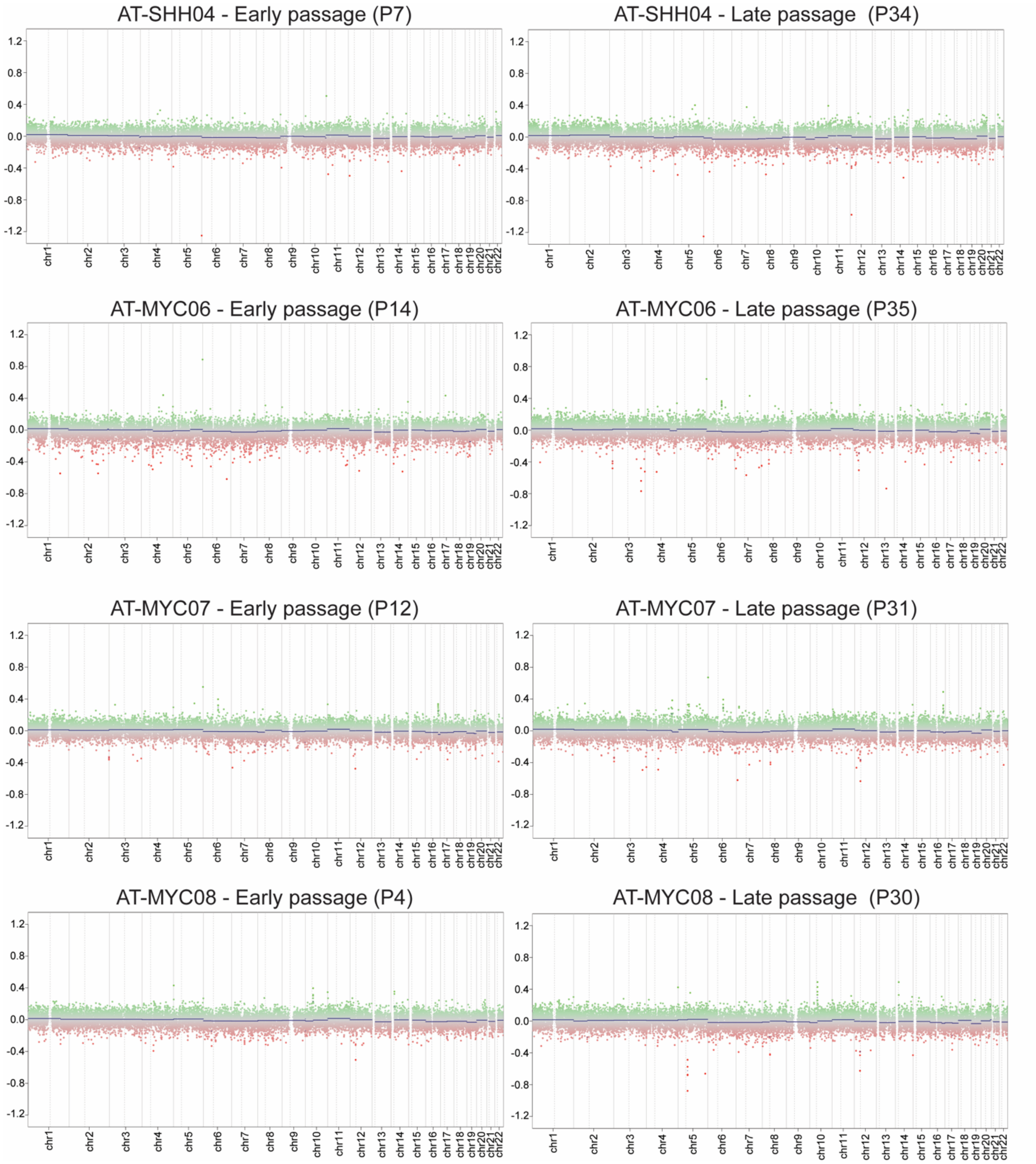
AT-SHH04 - Early passage (P7)
AT-SHH04 - Late passage (P34)
AT-MYC06 - Early passage (P14)
AT-MYC06 - Late passage (P35)
AT-MYC07 - Early passage (P12)
AT-MYC07 - Late passage (P31)
AT-MYC08 - Early passage (P4)
AT-MYC08 - Late passage (P30)
1.2
0.8
0.4
0.0
-0.4
-0.8
-1.2
chr1 chr2 chr3 chr4 chr5 chr6 chr7 chr8 chr9 chr10 chr11 chr12 chr13 chr14 chr15 chr16 chr17 chr18 chr19 chr20 chr21 chr22

Supplementary Figure S5

CNV plots generated by Conumee using 850K EPIC DNA methylation array of early and late passaged tumoroid models of AT-MYC06, AT-MYC07, AT-MYC08, and AT-SHH04. The log2 copy number ratio of tumoroid model versus reference brain tissue panel is plotted by chromosome. Dotted vertical lines indicate centromeres. Positive segments (gains) and negative segments (losses) are determined by comparison to the reference baseline.

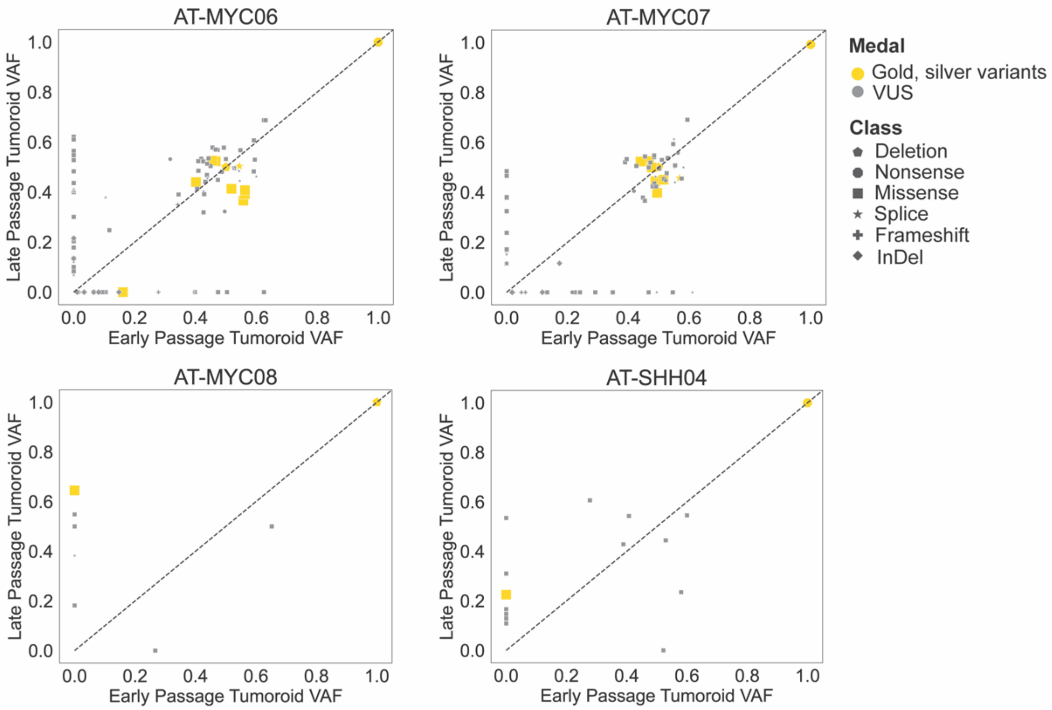
AT-MYC06
AT-MYC07
AT-MYC08
AT-SHH04
Late Passage Tumoroid VAF
Early Passage Tumoroid VAF
0.0
0.2
0.4
0.6
0.8
1.0
Medal
Gold, silver variants
VUS
Class
Deletion
Nonsense
Missense
Splice
Frameshift
InDel

Supplementary Figure S6

Variant allele frequency (VAF) plots of somatic variants from coding regions with evidence of clinical significance (gold variants), limited evidence (silver variants), or variants of uncertain levels of significance (VUS, including bronze and unknown variants as determined by Medal Ceremony). *SMARCB1* deletions which resulted in a single copy loss are plotted as 0.5 VAF; deletions which resulted in complete loss were plotted as 1.0 VAF. *SMARCB1* alterations in regions coinciding with a single copy loss or loss of heterozygosity are shown as 1.0 VAF.

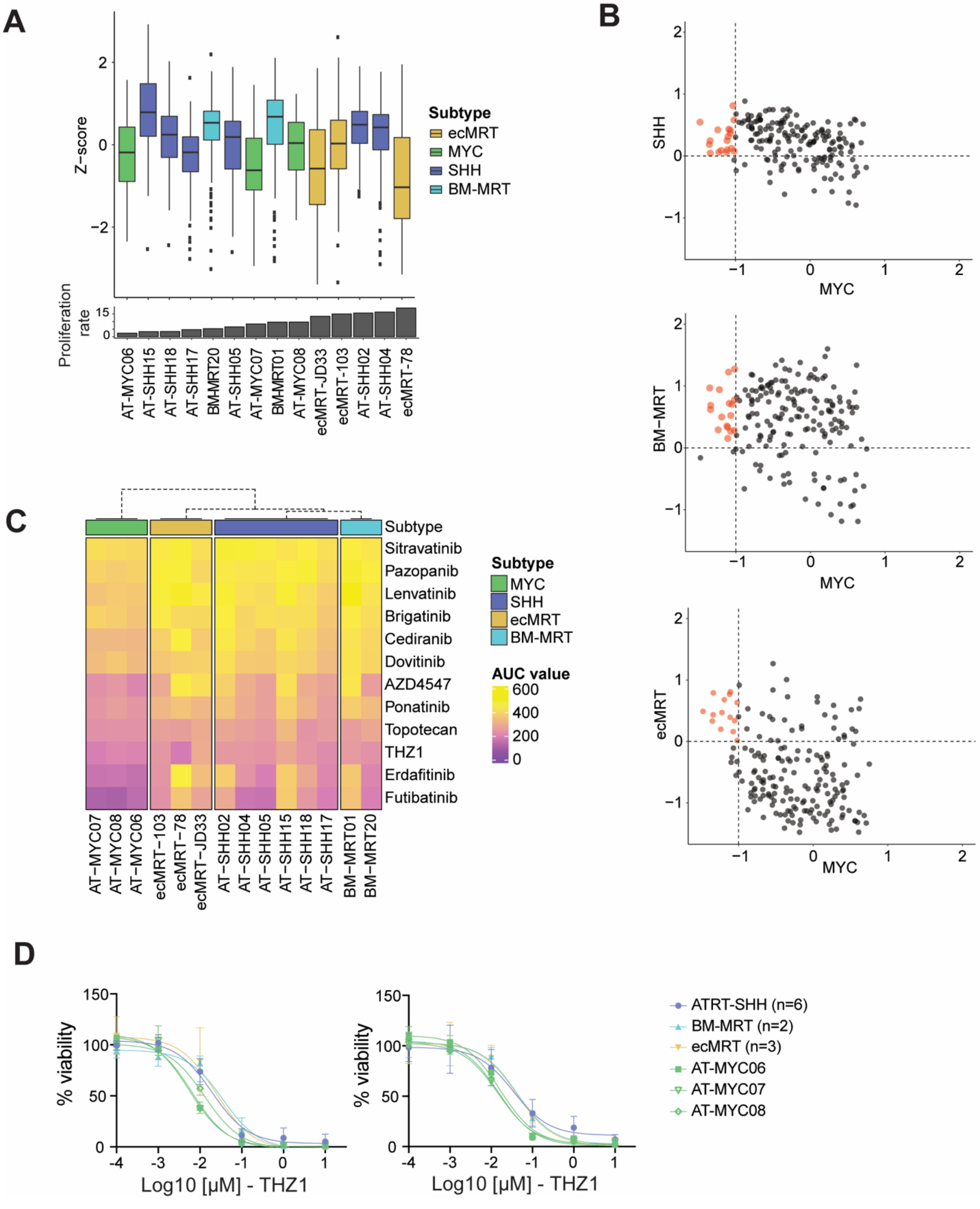

A
Z−score
Subtype
ecMRT
MYC
SHH
BM-MRT
Proliferation rate
AT-MYC06
AT-SHH15
AT-SHH18
AT-SHH17
BM-MRT20
AT-SHH05
AT-MYC07
BM-MRT01
AT-MYC08
ecMRT-JD33
ecMRT-103
AT-SHH02
AT-SHH04
ecMRT-78
B
SHH
MYC
BM−MRT
ecMRT
C
Subtype
Sitravatinib
Pazopanib
Lenvatinib
Brigatinib
Cediranib
Dovitinib
AZD4547
Ponatinib
Topotecan
THZ1
Erdafitinib
Futibatinib
AUC value
D
% viability
Log10 [μM] - THZ1
ATRT-SHH (n=6)
BM-MRT (n=2)
ecMRT (n=3)
AT-MYC06
AT-MYC07
AT-MYC08

Supplementary Figure S7

A Boxplot depicting z-score distribution of all 186 compounds per tumoroid model. Models are ordered based on their proliferation rate during the drug screen from left to right. **B** Scatterplot representation of the comparison made in Fig. 4C for each individual comparison. Dashed line indicates cut-off values. **C** Heatmap of the AUC values of the 12 drugs of interest. Dashed dendrogram indicates supervised clustering by subgroup. **D** Dose response curve of THZ1 and Topotecan, the two other DOIs identified as ATRT-MYC specific.

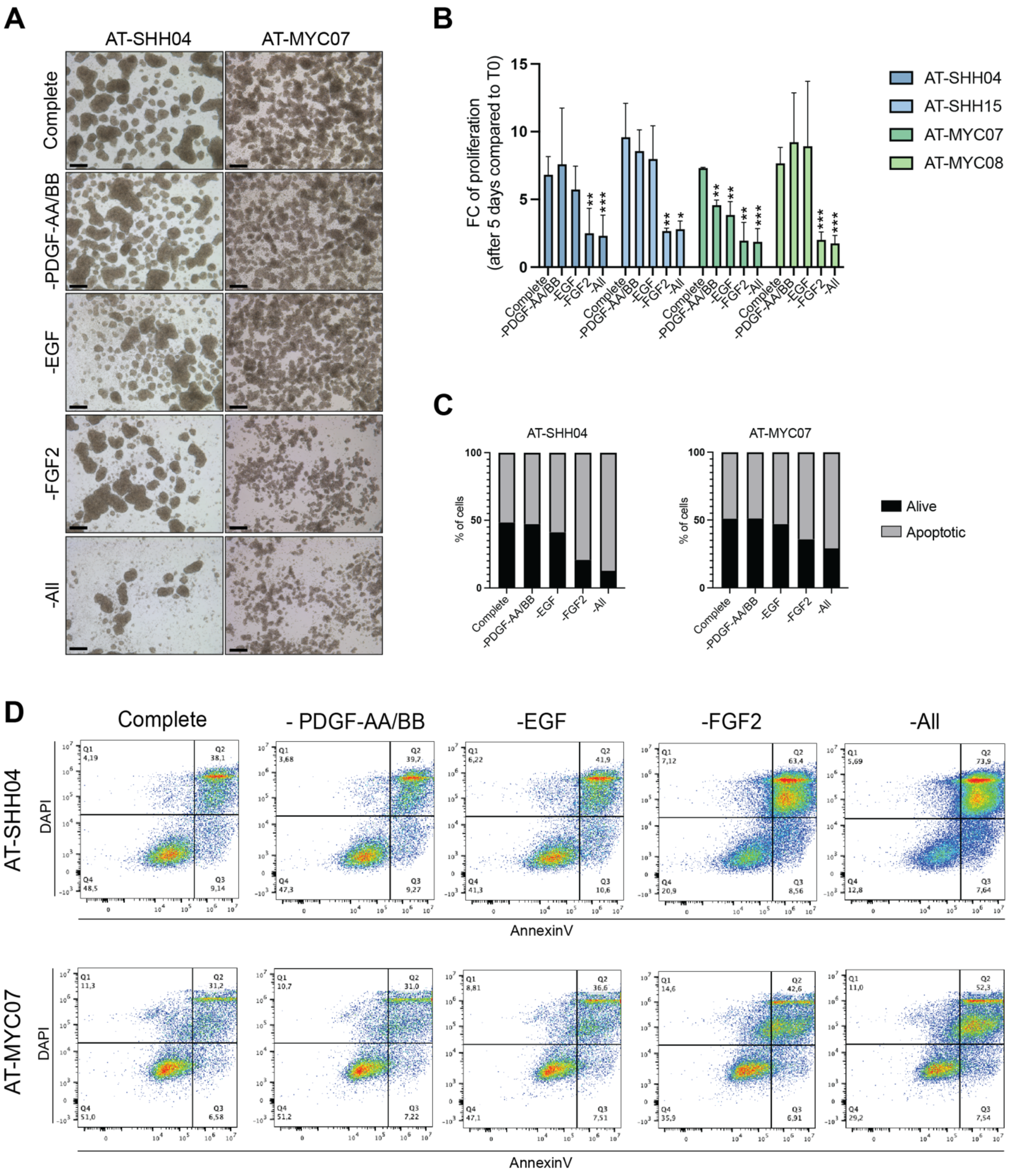
A
AT-SHH04
AT-MYC07
Complete
-PDGF-AA/BB
-EGF
-FGF2
-All
B
FC of proliferation (after 5 days compared to T0)
AT-SHH04
AT-SHH15
AT-MYC07
AT-MYC08
C
AT-SHH04
AT-MYC07
% of cells
Alive
Apoptotic
D
Complete
- PDGF-AA/BB
-EGF
-FGF2
-All
AT-SHH04
AT-MYC07
DAPI
AnnexinV

Supplementary Figure S8

A Brightfield images of medium depletion conditions for AT-SHH04 and AT-MYC07 after 5 days in each condition. Scale bars equal 100 µm. **B** Fold change in proliferation rate of 4 ATRT tumoroid models measured after 5 days by CellTiterGlo. Statistical significance was tested by paired t-test (*: p<= 0.05, **: p<= 0.01, ***: p<= 0.001.). **C** Quantification of apoptosis measured by AnnexinV/DAPI staining. **D** FACS gating of cell viability during growth factor withdrawal experiment. X-axis depicts AnnexinV signal and Y-axis DAPI signal. Percentage of alive cells are depicted in Q4.

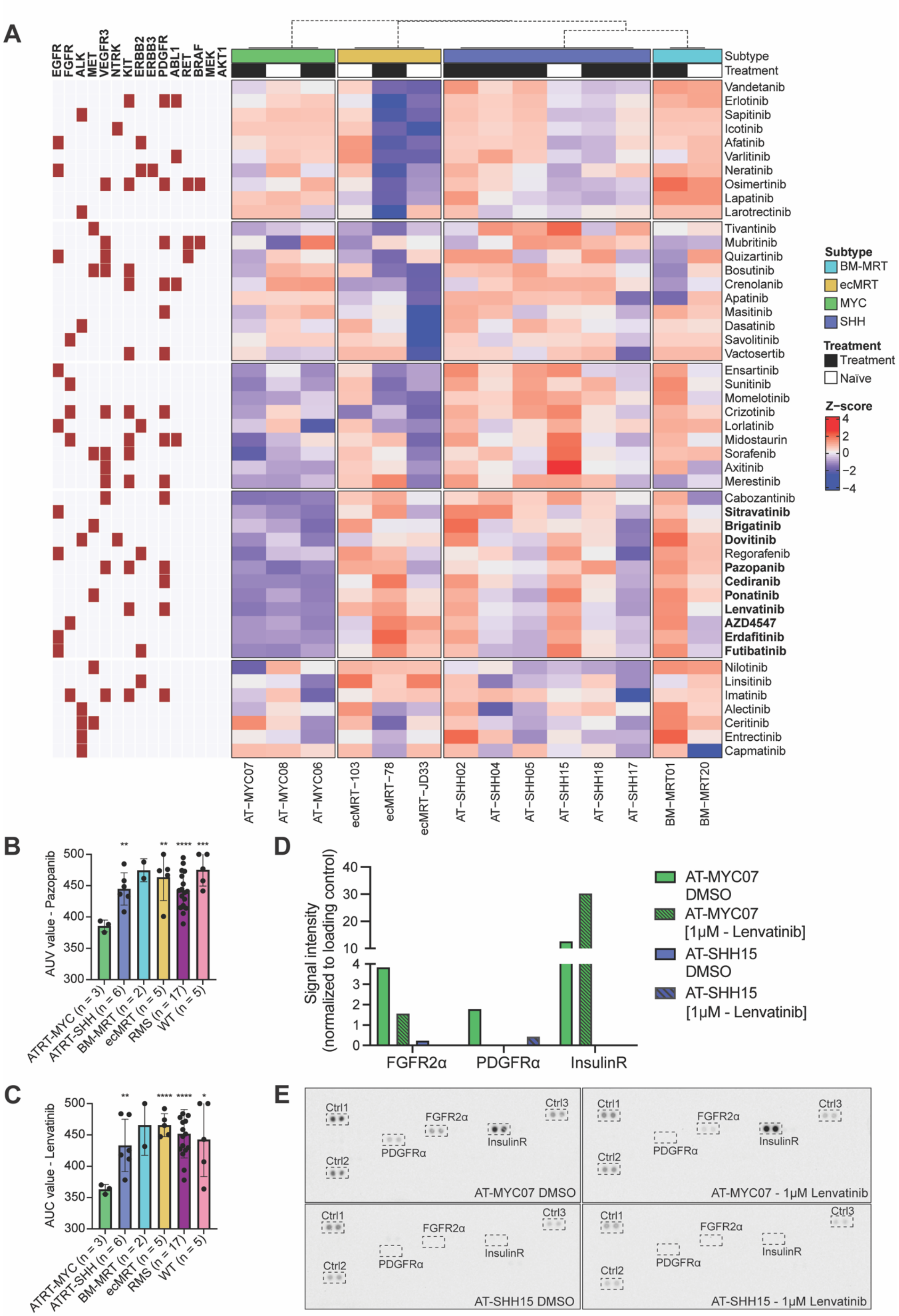

A
EGFR
FGFR
ALK
MET
VEGFR3
NTRK
KIT
ERBB2
ERBB3
PDGFR
ABL1
RET
BRAF
MEK
AKT1
Subtype
Treatment
Vandetanib
Erlotinib
Sapitinib
Icotinib
Afatinib
Varlitinib
Neratinib
Osimertinib
Lapatinib
Larotrectinib
Tivantinib
Mubritinib
Quizartinib
Bosutinib
Crenolanib
Apatinib
Masitinib
Dasatinib
Savolitinib
Vactosertib
Ensartinib
Sunitinib
Momelotinib
Crizotinib
Lorlatinib
Midostaurin
Sorafenib
Axitinib
Merestinib
Cabozantinib
Sitravatinib
Brigatinib
Dovitinib
Regorafenib
Pazopanib
Cediranib
Ponatinib
Lenvatinib
AZD4547
Erdafitinib
Futibatinib
Nilotinib
Linsitinib
Imatinib
Alectinib
Ceritinib
Entrectinib
Capmatinib
Subtype
BM-MRT
ecMRT
MYC
SHH
Treatment
Treatment
Naïve
Z-score
4
2
0
−2
−4
AT−MYC07
AT−MYC08
AT−MYC06
ecMRT−103
ecMRT−78
ecMRT−JD33
AT−SHH02
AT−SHH04
AT−SHH05
AT−SHH15
AT−SHH18
AT−SHH17
BM−MRT01
BM−MRT20
B
AUV value - Pazopanib
500
450
400
350
300
**
**
****
***
ATRT-MYC (n = 3)
ATRT-SHH (n = 6)
BM-MRT (n = 2)
ecMRT (n = 5)
RMS (n = 17)
WT (n = 5)
C
AUC value - Lenvatinib
500
450
400
350
300
**
****
****
*
ATRT-MYC (n = 3)
ATRT-SHH (n = 6)
BM-MRT (n = 2)
ecMRT (n = 5)
RMS (n = 17)
WT (n = 5)
D
Signal intensity
(normalized to loading control)
40
30
20
10
4
3
2
1
0
FGFR2α
PDGFRα
InsulinR
AT-MYC07
DMSO
AT-MYC07
[1µM - Lenvatinib]
AT-SHH15
DMSO
AT-SHH15
[1µM - Lenvatinib]
E
Ctrl1
Ctrl2
Ctrl3
FGFR2α
PDGFRα
InsulinR
AT-MYC07 DMSO
AT-MYC07 - 1µM Lenvatinib
AT-SHH15 DMSO
AT-SHH15 - 1µM Lenvatinib

Supplementary Figure S9

A Overview of z-scores of the 48 multi kinase inhibitors present in the drug library. Dashed dendrogram indicates supervised clustering by subgroup. Left panel of the heatmap shows the targets of the different inhibitors (target is indicated by red color). Row clusters were made by k-mean clustering. Top hits for ATRT-MYC tumoroids are indicated in bold. **B, C** Comparison of average AUC value of Pazopanib (B) and Lenvatinib (C) of multiple different tumor entities (RMS = Rhabdomyosarcoma, WT = Wilm's Tumor). Statistical significance was tested by Welch's t-test against the ATRT-MYC group (*: *p*<= 0.05, **: *p*<= 0.01, ***: *p*<= 0.001, ****: *p*<=0.0001.) Value is missing for BM-MRT due to small sample size. **D** Quantification of phospho-RTK array. Signal intensity of each two dots per RTK was normalized to loading controls per blot. **E** Dot blot images of the phospho-RTK array. Dashed squares indicate quantified signals. Images were taken simultaneously with the same exposure time.

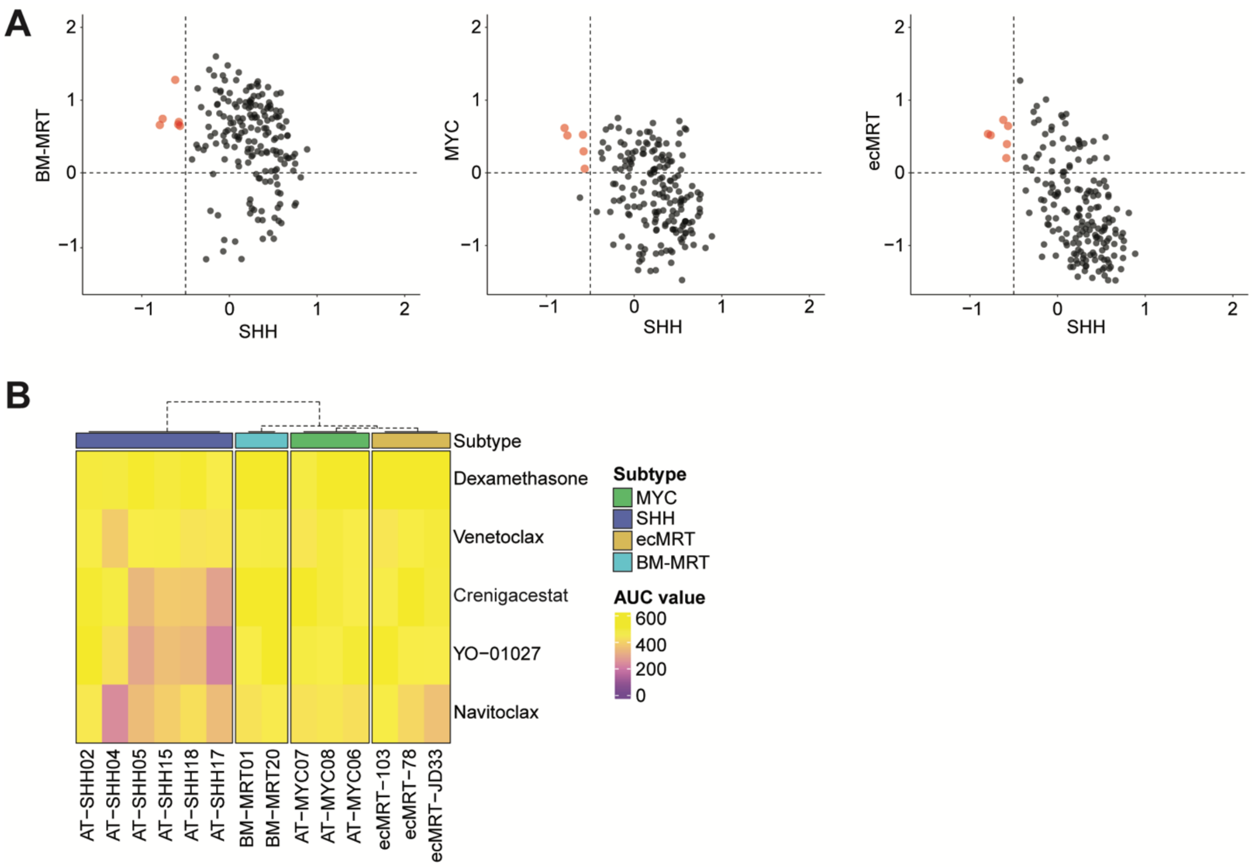
A
BM-MRT
MYC
ecMRT
SHH
B
Subtype
Dexamethasone
Venetoclax
Crenigacestat
YO−01027
Navitoclax
Subtype
MYC
SHH
ecMRT
BM-MRT
AUC value
600
400
200
0
AT−SHH02
AT−SHH04
AT−SHH05
AT−SHH15
AT−SHH18
AT−SHH17
BM−MRT01
BM−MRT20
AT−MYC07
AT−MYC08
AT−MYC06
ecMRT−103
ecMRT−78
ecMRT−JD33

Supplementary Figure S10

A Scatterplots representing comparisons made between SHH and other tumor entities. Dashed line indicates cut-off values. Red dots are compounds considered for comparison. **B** AUC values of the five drugs of interest. Dashed dendrogram indicates supervised clustering by subgroup.

Legends Supplementary Tables

Supplementary Table 1

Overview of SNVs, CNVs and InDels identified in ATRT tumoroid models and matching primary samples identified by WES or WGS. (T = tumoroid; X = xenograft; D = patient sample, G = germline)

Supplementary Table 2

Pearson R correlation scores of inter-sample comparisons (per model) or between matching tumoroid-PDOX pairs (T = tumoroid; X = xenograft; D = patient sample; TLP = tumoroid late passage).

Supplementary Table 3

GSA analysis on differential CpG probes of multiple different comparisons (T = tumoroid; X = xenograft; D = patient sample; TLP = tumoroid late passage).

Supplementary Table 4

Molecular Neuropathology (MNP) classifier scores for the tumoroids and parental samples (T = tumoroid; X = xenograft)

Supplementary Table 5

Composition of the drug library used for the drug screens.

Supplementary Table 6

IC_{50} values [log10 µM] of all identified drugs of interest (DOIs). N/A: not applicable (i.e., compounds not reaching an IC_{50} value).
